# Prevalence of the Metabolic Syndrome in Latin America and its association with sub-clinical carotid atherosclerosis: the CARMELA cross sectional study

**DOI:** 10.1186/1475-2840-8-52

**Published:** 2009-09-26

**Authors:** Jorge Escobedo, Herman Schargrodsky, Beatriz Champagne, Honorio Silva, Carlos P Boissonnet, Raul Vinueza, Marta Torres, Rafael Hernandez, Elinor Wilson

**Affiliations:** 1 Medical Research Unit on Clinical Epidemiology, Mexican Social Security Institute, Mexico City, Mexico; 2 Gabriel Mancera 222m Col. Del Valle, 03100 Mexico City, Mexico; 3 Department of Cardiology, Hospital Italiano de Buenos Aires, Buenos Aires, Argentina; 4 InterAmerican Heart Foundation, Dallas, Texas, USA; 5 InterAmerican Foundation for Clinical Research, New York, New York, USA; 6 Coronary Care Unit, Centro de Educación Médica e Investigaciones Clínicas "Norberto Quirno," Buenos Aires, Argentina; 7 Clinical Protocol Manager Canada/Latin America/Africa/Middle East Region Worldwide Development Operations, Pfizer Inc., New York, New York, USA; 8 Programa Buenos Aires de Control de Calidad Externo-CEMIC, Buenos Aires, Argentina; 9 Clinical Pharmacology Unit and Hypertension Clinic, School of Medicine, Universidad Centroccidental "Lisandro Alvarado," Decanato de Medicina, Barquisimeto, Venezuela; 10 Department of Community and Preventive Health, School of Medicine & Dentistry, University of Rochester, Rochester, New York, USA

## Abstract

**Background:**

Metabolic syndrome increases cardiovascular risk. Limited information on its prevalence in Latin America is available. The Cardiovascular Risk Factor Multiple Evaluation in Latin America (CARMELA) study included assessment of metabolic syndrome in 7 urban Latin American populations.

**Methods:**

CARMELA was a cross-sectional, population-based, observational study conducted in Barquisimeto, Venezuela; Bogota, Colombia; Buenos Aires, Argentina; Lima, Peru; Mexico City, Mexico; Quito, Ecuador; and Santiago, Chile. The prevalence of metabolic syndrome, defined according to the National Cholesterol Education Program Adult Treatment Panel III (NCEP ATP III), and associated carotid atherosclerosis were investigated in 11,502 participants aged 25 to 64 years.

**Results:**

Across CARMELA cities, metabolic syndrome was most prevalent in Mexico City (27%) and Barquisimeto (26%), followed by Santiago (21%), Bogota (20%), Lima (18%), Buenos Aires (17%), and Quito (14%). In nondiabetic participants, prevalence was slightly lower but followed a comparable ranking. Overall, 59%, 59%, and 73% of women with high triglycerides, hypertension, or glucose abnormalities, respectively, and 64%, 48% and 71% of men with abdominal obesity, hypertension, or glucose abnormalities, respectively, had the full metabolic syndrome. Prevalence of metabolic syndrome increased with age, markedly so in women. Mean common carotid artery intima-media thickness (CCAIMT) and prevalence of carotid plaque increased steeply with increasing numbers of metabolic syndrome components; mean CCAIMT was higher and plaque more prevalent in participants with metabolic syndrome than without.

**Conclusion:**

The prevalence of metabolic syndrome and its components by NCEP ATP III criteria was substantial across cities, ranging from 14% to 27%. CARMELA findings, including evidence of the association of metabolic syndrome and carotid atherosclerosis, should inform appropriate clinical and public health interventions.

## Background

The concept of the metabolic syndrome has developed in stages over the past 80 years; it is now recognized that the combination of abdominal obesity, glucose metabolism abnormalities, hypertension, and dyslipidemia, accompanied by prothrombotic and proinflammatory states, leads to type 2 diabetes and vascular diseases, including coronary heart disease and stroke [1,2].

Metabolic syndrome predicts total, cardiovascular, and coronary heart disease mortality; in fact, the presence of even 1 or 2 components of the metabolic syndrome increases overall mortality compared with the absence of any component of the metabolic syndrome [3-6]. A 65% excess risk has been estimated for cardiovascular disease in individuals with the metabolic syndrome [7]. Metabolic syndrome even predicts the occurrence of sudden death, independent of the presence of other cardiovascular risk factors [8]. Metabolic syndrome also predicts the incidence and progression of carotid atherosclerosis [9].

As reviewed recently, worldwide prevalence of metabolic syndrome ranges from <10% to as much as 84%, depending on age, region, urban or rural environment, ethnicity, and the definition of metabolic syndrome used [10-12]. In the United States, between 1994 and 2000, prevalence of metabolic syndrome in adults increased from 23% to 27% along with an increase in obesity and physical inactivity [13]. Developing regions like Latin America, undergoing sea change in the lifestyle factors contributing to the metabolic syndrome, may see even greater increases in prevalence over a relatively short span of time. Local public health and clinical efforts to stem morbidity and mortality from the metabolic syndrome must be based on the relevant local prevalence of its components. The Cardiovascular Risk Factor Multiple Evaluation in Latin America (CARMELA) study investigated risk factors for cardiovascular disease in 7 Latin American cities, as reported elsewhere [14]. With cardiovascular risk and clinical interventions in mind, the National Cholesterol Education Program Adult Treatment Panel III (NCEP ATP III) criteria [2] were used to assess metabolic syndrome prevalence in CARMELA cities. The association of metabolic syndrome with mean common carotid artery intima-media thickness (CCAIMT), as a marker of carotid atherosclerosis, was also investigated.

## Methods

### Study Design

CARMELA was a multistage, cross-sectional epidemiologic study conducted between September 2003 and August 2005, in Barquisimeto, Venezuela; Bogota, Colombia; Buenos Aires, Argentina; Lima, Peru; Mexico City, Mexico; Quito, Ecuador; and Santiago, Chile. The study was conducted according to the Declaration of Helsinki and the Guides for Good Clinical Practice. Approximately 1,600 participants per city between the ages of 25 and 64 years were included, stratified by sex and age (10-year groups). The study was approved by an institutional review committee in all participating cities, and all subjects gave informed consent. Study methods are described in detail elsewhere [14].

### Anthropometry

Waist circumference was measured at the midpoint between the last rib and the iliac crest with participants standing and wearing only undergarments.

### Blood Glucose and Lipids

Participants were asked to refrain from using laxatives containing glycerin for 48 hours and from consuming glycerin-containing products and other sweets for 24 hours prior to blood sampling. Blood was drawn in the fasting state; only water, black coffee, or unsweetened tea and medications other than antidiabetic medications were permitted during the 12 hours prior to sampling. Following the sampling, participants were allowed to resume their usual antidiabetic medication. Plasma glucose was assayed within 6 hours. Serum was assayed for high density lipoprotein cholesterol and triglycerides.

### Blood Pressure

Blood pressure (at rest) was measured with the participant seated. Two readings were taken 5 minutes apart; if different by >5 mm Hg, measurements were repeated until 2 concordant readings were obtained.

### Measurement of CCAIMT

Far wall CCAIMT was evaluated according to the Mannheim intima-media thickness consensus [15]. Both common carotid arteries were examined by B-mode ultrasonography with participants in the supine position, using phased array 7.5 MHz transducers and ultrasound apparatus no older than 7 years. M'AthStd^® ^software (Intelligence in Medical Technologies, Paris, France) automatically measured the CCAIMT; measurements were taken over a 10 mm length and averaged, and quality of acquisition was evaluated. Data were analyzed at a central laboratory at Intelligence in Medical Technologies, Paris, France.

### Definitions

Diabetes was defined as a fasting plasma glucose level ≥ 7.0 mmol/l (126 mg/dL) [16] or self-reported diabetes. Metabolic syndrome was defined by NCEP ATP III criteria as the presence of 3 or more of the following: waist >102 cm in men, >88 cm in women; triglycerides ≥ 1.70 mmol/l (150 mg/dL); high density lipoprotein cholesterol (HDL-C) <1.03 mmol/l (40 mg/dL) in men, <1.29 mmol/l (50 mg/dL) in women; blood pressure ≥ 130/85 mm Hg; and fasting plasma glucose ≥ 6.11 mmol/l (110 mg/dL) or self-reported diabetes [2].

### Statistical Analysis

Statistical processing addressed the nonequal probability character of the sample and the structure of the design to generate data adjusted for the age and sex distribution of the population of each city. Means and prevalence along with their 95% confidence intervals were estimated by survey analysis procedures (SAS Software, Release 9.1, Cary, North Carolina, USA), taking into account the multistage stratified sampling design via CLUSTER and STRATA statements.

## Results

A total of 11,550 participants between the ages of 25 and 64 years were enrolled in CARMELA between September 2003 and August 2005. Data for 11,502 participants were evaluable for metabolic syndrome: Barquisimeto, n = 1,836; Bogotá, n = 1,550; Buenos Aires, n = 1,476; Lima, n = 1,645; Mexico City, n = 1,720; Quito, n = 1,627; and Santiago, n = 1,648. Per the study design, age and sex distribution was relatively even for each city.

### Prevalence of the Metabolic Syndrome in the Overall CARMELA Population

Metabolic syndrome was most prevalent in Mexico City (27%) and Barquisimeto (26%), followed by Santiago (21%) and Bogota (20%); lower prevalence was found in Lima (18%), Buenos Aires (17%), and Quito (14%). Overall, metabolic syndrome was more prevalent in women than men (22% vs 20%, respectively), with the exception of Buenos Aires and Barquisimeto where more men than women had metabolic syndrome. As expected, the prevalence of metabolic syndrome increased with age. In all cities, women showed markedly greater increase in metabolic syndrome with increasing age than men; in the oldest age group, female prevalence (range 25% to 49%) was greater than male prevalence (range 13% to 38%) in all cities except Buenos Aires (See Table 1 and Figure S1 in additional file 1). Of components of metabolic syndrome, the abdominal obesity was notably more prevalent in women than men, and the difference was accentuated in successively older age groups.

**Table 1:** Prevalence (%) (95% confidence interval) in the nondiabetic population* of the Metabolic Syndrome, according to NCEP ATP III.

	**Age group**				
	**25 - 34**	**35 - 44**	**45 - 54**	**55 - 64**	**Overall prevalence**
Barquisimeto					
Men	19.7 (13.1-26.2)	27.2 (20.7-33.7)	32.4 (25.5-39.4)	38.1 (30.7-45.5)	23.0 (19.1-27.0)
Women	11.6 (7.2-16.0)	23.9 (19.2-28.6)	37.7 (32.3-43.1)	47.8 (41.8-53.8)	22.7 (20.0-25.3)
Bogota					
Men	10.7 (6.2-15.1)	18.5 (13.1-23.8)	25.9 (20.4-31.4)	30.1 (22.9-37.3)	14.7 (11.8-17.5)
Women	8.3 (4.6-11.9)	16.8 (10.8-22.7)	36.2 (30.2-42.3)	48.6 (41.8-55.5)	18.2 (15.5-20.9)
Buenos Aires					
Men	11.8 (7.2-16.4)	21.8 (15.7-27.9)	26.6 (20.3-32.9)	33.0 (26.0-40.0)	17.3 (14.8-19.8)
Women	3.3 (0.8-5.9)	7.5 (3.6-11.4)	16.9 (10.4-23.5)	24.6 (17.8-31.4)	9.7 (7.1-12.3)
Lima					
Men	10.0 (5.9-14.2)	15.8 (9.0-22.5)	19.8 (14.1-25.4)	25.1 (18.2-32.1)	13.2 (10.6-15.8)
Women	6.4 (3.2- 9.7)	18.7 (12.9-24.4)	34.8 (28.8-40.7)	36.8 (29.5-44.0)	17.6 (15.0-20.1)
Mexico City					
Men	22.2 (15.6-28.8)	22.6 (17.7-27.6)	33.5 (28.0-39.0)	37.2 (30.6-43.7)	22.4 (18.9-25.8)
Women	16.5 (11.9-21.1)	27.4 (20.3-34.5)	35.5 (30.0-40.9)	49.3 (42.4-56.1)	22.2 (19.1-25.4)
Quito					
Men	3.6 (0.7- 6.5)	6.3 (3.5- 9.1)	12.9 (8.2-17.6)	13.4 (8.6-18.2)	5.5 (3.9-7.1)
Women	7.8 (3.6-12.1)	18.0 (11.8-24.2)	35.5 (29.3-41.6)	37.4 (31.1-43.7)	16.4 (13.5-19.3)
Santiago					
Men	9.9 (5.8-14.0)	17.5 (12.0-23.0)	27.0 (20.8-33.1)	31.5 (25.5-37.4)	15.3 (12.8-17.9)
Women	11.6 (7.3-15.9)	22.3 (16.0-28.6)	26.1 (20.4-31.7)	42.4 (36.4-48.5)	19.0 (16.0-22.0)

*CARMELA participants excluding participants with fasting plasma glucose ≥ 7.0 mmol/l (126 mg/dL) and/or self-reported diabetes.

### Prevalence of Specific Components of the Metabolic Syndrome

Table 2 reports the prevalence of each component of the metabolic syndrome within the population of all participants with metabolic syndrome (with or without a history of diabetes mellitus). Overall, 86% had high triglycerides, 86% had low HDL-C, and 60% were hypertensive. Across cities, abdominal obesity was present in 81% to 93% of women, and in 46% to 73% of men with metabolic syndrome.

**Table 2:** Prevalence* and 95% confidence interval of specific components of the metabolic syndrome in population with metabolic syndrome

	**Abdominal obesity >102 cm in men or >88 cm in women**	**Triglycerides ≥ 1.70 mmol/l (150 mg/dL)**	**HDL-C <1.03 mmol/l (40 mg/dL) in men or <1.29 mmol/l (50 mg/dL) in women**	**Blood pressure ≥ 130/85 mm Hg or treatment**	**Glucose abnormalities^†^**
Overall	76.3 (74.0-78.6)	85.9 (84.3-87.6)	85.6 (83.8-87.4)	60.1 (57.7-62.5)	31.2 (28.9-33.2)
Men	61.3 (57.2-65.5)	90.2 (88.2-92.1)	82.7 (80.2-85.2)	68.0 (64.7-71.3)	32.6 (29.5-35.7)
Women	88.1 (86.2-90.0)	82.6 (80.2-85.0)	87.9 (85.9-90.0)	53.8 (50.8-56.9)	30.1 (27.1-33.0)
Barquisimeto					
Men	56.6 (49.1-64.1)	90.3 (85.9-94.8)	92.9 (89.6-96.2)	82.2 (76.7-87.8)	23.1 (17.4-28.8)
Women	84.0 (79.6-88.3)	78.7 (73.9-83.5)	96.4 (94.8-98.0)	63.6 (57.3-69.9)	19.4 (15.5-23.3)
Bogota					
Men	45.8 (35.6-56.1)	92.1 (87.5-96.8)	92.9 (89.1-96.8)	57.6 (48.8-66.3)	40.6 (32.1-49.2)
Women	86.0 (81.5-90.5)	83.3 (78.3-88.2)	93.7 (90.2-97.2)	49.8 (42.2-57.4)	28.6 (22.2-34.9)
Buenos Aires					
Men	73.1 (65.6-80.5)	77.5 (71.1-84.0)	67.7 (60.2-75.2)	93.2 (89.5-96.8)	31.8 (24.0-39.7)
Women	88.9 (82.9-94.9)	65.4 (56.7-74.0)	79.9 (72.4-87.3)	77.3 (68.3-86.4)	31.1 (21.3-40.9)
Lima					
Men	55.3 (46.5-64.2)	87.3 (81.8-92.7)	93.4 (89.0-97.7)	70.5 (62.9-78.0)	30.7 (22.0-39.5)
Women	80.8 (75.2-86.3)	82.6 (76.9-88.4)	97.9 (95.8-100.0)	51.7 (44.3-59.1)	24.0 (18.6-29.3)
Mexico City					
Men	72.7 (65.9-79.5)	93.0 (89.7-96.3)	78.0 (73.7-83.9)	58.3 (52.8-63.7)	30.4 (24.5-36.2)
Women	93.1 (90.6-95.5)	85.7 (80.0-91.3)	77.7 (73.2-82.1)	52.8 (47.3-58.2)	35.2 (27.9-42.6)
Quito					
Men	48.8 (36.3-61.4)	97.9 (94.8-101.0)	83.9 (75.8-92.1)	62.3 (48.1-76.5)	33.4 (22.5-44.3)
Women	90.1 (85.5-94.7)	83.3 (79.0-87.7)	92.0 (88.9-95.1)	37.0 (28.7-45.2)	32.6 (26.1-39.1)
Santiago					
Men	60.7 (51.9-69.5)	93.0 (89.6-96.3)	73.5 (66.4-80.7)	76.6 (69.1-84.0)	31.4 (24.9-37.9)
Women	90.6 (86.7-94.4)	82.9 (77.6-88.2)	86.6 (81.9-91.2)	57.1 (50.6-63.6)	31.1 (24.1-38.1)

*Prevalence among participants with metabolic syndrome, with or without previously diagnosed diabetes mellitus.

^†^Participants with fasting plasma glucose >6.11 mmol/l (110 mg/dL) or previous diagnosis of diabetes mellitus.

### Prevalence of the Metabolic Syndrome in Participants With Specific Components

Prevalence of the metabolic syndrome in subsets of the overall population with specific components of the syndrome is reported in Table 3. Overall, approximately two-thirds of men with abdominal obesity or glucose abnormalities, and approximately 60% to 70% of women with high triglycerides, hypertension, or glucose abnormalities had the full metabolic syndrome. In all cities except Mexico City, the prevalence of metabolic syndrome among participants with low HDL-C was fairly low. Prevalence of the syndrome among women with high triglycerides was notably higher than among men in all cities except Barquisimeto and Buenos Aires.

**Table 3:** Prevalence (%)(95% confidence interval) of the metabolic syndrome among subsets of participants* with each of the 5 NCEP ATP III risk factor components of the syndrome

	**Abdominal obesity >102 cm in men or >88 cm in women**	**Triglycerides ≥ 1.70 mmol/l (150 mg/dL)**	**HDL-C <1.03 mmol/l (40 mg/dL) in men or <1.29 mmol/l (50 mg/dL) in women**	**Blood pressure ≥ 130/85 mm Hg or treatment**	**Glucose abnormalities**^†^
Overall	53.3 (51.0-55.5)	45.7 (43.9-47.5)	34.6 (33.0-36.2)	52.5 (50.3-54.7)	71.6 (68.3-74.8)
Men	64.0 (60.3-67.7)	36.2 (34.0-38.4)	35.5 (33.0-38.0)	47.5 (44.7-50.2)	70.5 (66.4-74.7)
Women	48.7 (45.9-51.6)	59.0 (56.2-61.9)	34.0 (32.0-36.0)	58.7 (55.6-61.8)	72.5 (67.7-77.2)
Barquisimeto					
Men	75.7 (66.9-84.5)	50.3 (44.5-56.1)	34.5 (29.1-39.9)	60.2 (53.7-66.7)	82.1 (72.9-91.2)
Women	61.1 (55.9-66.3)	66.9 (62.6-71.2)	32.4 (28.5-36.3)	59.8 (54.1-65.5)	70.3 (59.4-81.2)
Bogota					
Men	77.1 (68.5-85.7)	32.1 (27.7-36.5)	29.4 (25.2-33.7)	53.1 (45.9-60.2)	71.1 (60.3-81.9)
Women	55.0 (49.0-60.9)	55.0 (48.4-61.6)	28.5 (25.2-31.9)	64.8 (56.2-73.4)	61.6 (50.1-73.1)
Buenos Aires					
Men	56.6 (50.3-62.8)	57.2 (49.9-64.5)	50.1 (43.3-57.0)	38.4 (34.2-42.5)	70.8 (60.1-81.6)
Women	36.0 (29.7-42.2)	62.3 (53.1-71.4)	31.7 (25.1-38.3)	31.7 (25.1-38.4)	68.6 (55.3-81.9)
Lima					
Men	73.7 (65.0-82.4)	35.1 (29.4-40.7)	22.9 (18.9-26.8)	46.4 (39.1-53.6)	74.5 (63.2-85.8)
Women	50.9 (44.3-57.5)	61.5 (55.2-67.8)	24.3 (21.1-27.5)	64.1 (56.7-71.5)	76.6 (65.7-87.4)
Mexico City					
Men	59.6 (52.4-66.8)	38.3 (33.6-43.0)	58.8 (51.7-65.9)	61.8 (55.2-68.5)	71.1 (64.7-77.4)
Women	46.7 (40.2-53.2)	59.3 (53.5-65.1)	53.0 (47.1-58.9)	67.7 (60.3-75.1)	79.5 (71.6-87.5)
Quito					
Men	66.2 (52.3-80.1)	14.5 (11.1-17.9)	22.6 (16.9-28.2)	34.1 (25.7-42.5)	44.5 (31.8-57.2)
Women	46.2 (40.9-51.5)	51.3 (42.9-59.7)	38.6 (32.8-44.4)	62.2 (53.7-70.7)	71.9 (62.1-81.7)
Santiago					
Men	61.1 (52.6-69.7)	36.4 (31.5-41.3)	44.8 (38.4-51.1)	39.5 (34.1-44.8)	68.1 (58.8-77.3)
Women	47.8 (42.6-52.9)	61.9 (56.1-67.8)	43.3 (37.8-48.9)	57.6 (51.7-63.5)	74.9 (65.4-84.5)

*All participants, with or without diabetes mellitus, were grouped by presence of specific components, and prevalence of metabolic syndrome within each group is reported here.

^†^All participants with fasting plasma glucose >6.11 mmol/l (110 mg/dL) or previous diagnosis of diabetes mellitus.

### Prevalence of Metabolic Syndrome Among Participants Without Diabetes

Table 4 reports the prevalence of the number of components of the metabolic syndrome among CARMELA participants without a history of diabetes mellitus. In all cities, >60% of the participants had at least 1 component of the metabolic syndrome. As in the overall CARMELA population, the prevalence of metabolic syndrome in the nondiabetic population was higher in women than men, except in Barquisimeto and Buenos Aires.

**Table 4:** Prevalence (%) (95% confidence interval) in the nondiabetic population* of 1 to 5 of the NCEP ATP III risk factor components of the metabolic syndrome and diagnosis of metabolic syndrome

	**Number of Components**					
	**0**	**1**	**2**	**3**	**4**	**5**
Overall	23.4 (22.3-24.5)	32.8 (31.7-34.0)	26.5 (25.4-27.6)	13.0 (12.2-13.8)	4.0 (3.6-4.4)	0.3 (0.2-0.4)
Men	24.6 (23.1-26.2)	30.8 (29.1-32.4)	28.5 (26.9-30.0)	12.2 (11.1-13.4)	3.6 (3.0-4.1)	0.3 (0.1-0.5)
Women	22.3 (20.8-23.8)	34.7 (33.0-36.3)	24.8 (23.3-26.2)	13.7 (12.5-14.8)	4.3 (3.8-4.9)	0.3 (0.1-0.4)
Barquisimeto						
Men	14.1 (11.2-16.7)	32.6 (28.3-37.0)	30.3 (26.5-34.1)	15.8 (12.8-18.8)	6.7 (4.7-8.8)	0.5 (0.0-1.0)
Women	13.4 (10.9-16.0)	37.5 (33.8-41.2)	26.4 (23.4-29.5)	16.3 (14.0-18.5)	6.2 (4.7-7.8)	0.2 (0.0-0.3)
Bogota						
Men	21.6 (18.1-25.0)	30.7 (26.6-34.9)	33.0 (29.4-36.6)	11.4 (8.6-14.2)	3.0 (1.8-4.1)	0.3 (0.0-0.7)
Women	17.6 (14.3-20.9)	35.4 (31.9-38.9)	28.8 (25.5-32.1)	14.0 (11.2-16.8)	4.0 (2.9-5.2)	0.2 (0.0-0.4)
Buenos Aires						
Men	28.5 (25.1-31.9)	30.2 (27.3-33.1)	24.0 (20.4-27.5)	12.7 (10.5-15.0)	4.4 (2.9-5.9)	0.2 (0.0-0.5)
Women	37.7 (33.8-41.5)	36.7 (32.8-40.6)	15.9 (13.0-18.8)	7.5 (5.4-9.6)	2.1 (0.8-3.3)	0.1 (0.0-0.4)
Lima						
Men	21.5 (18.0-25.0)	35.8 (32.0-39.7)	29.5 (26.0-33.0)	9.7 (7.5-11.8)	3.2 (1.9-4.5)	0.3 (0.0-0.6)
Women	12.1 (9.5-14.7)	44.2 (40.4-48.1)	26.1 (22.7-29.5)	13.2 (10.9-15.3)	4.2 (3.0-5.5)	0.2 (0.0-0.5)
Mexico City						
Men	23.7 (20.5-27.0)	26.0 (23.4-28.5)	27.9 (24.7-31.1)	17.3 (14.1-20.5)	4.6 (3.2-6.0)	0.5 (0.0-1.0)
Women	24.3 (20.7-27.8)	27.5 (24.1-31.0)	26.0 (22.9-29.0)	16.7 (14.0-19.4)	5.0 (3.7-6.3)	0.5 (0.0-1.0)
Quito						
Men	39.2 (34.8-43.5)	32.5 (28.9-36.0)	22.8 (19.8-25.9)	4.3 (2.9-5.8)	1.1 (0.4-1.8)	0.1 (0.0-0.2)
Women	30.5 (26.9-34.1)	30.9 (27.2-34.5)	22.2 (18.8-25.7)	13.4 (10.8-16.1)	2.7 (1.7-3.6)	0.3 (0.0-0.6)
Santiago						
Men	28.6 (24.5-32.6)	29.8 (25.6-33.9)	26.3 (22.7-29.9)	11.7 (9.5-14.1)	3.3 (2.0-4.5)	0.3 (0.0-0.7)
Women	28.6 (24.8-32.3)	30.5 (26.9-34.2)	21.9 (19.1-24.8)	13.3 (10.8-15.8)	5.5 (3.8-7.1)	0.2 (0.0-0.5)

*CARMELA participants excluding participants with fasting plasma glucose ≥ 7.0 mmol/l (126 mg/dL) and/or self-reported diabetes.

### Metabolic Syndrome and Carotid Atherosclerosis

In both men and women overall, mean CCAIMT increased steeply with increasing numbers of metabolic syndrome components (Table 5 and Figure S2 in additional file 1). Similarly, the prevalence of carotid plaque also increased with increasing numbers of components and overall, was approximately 50% more prevalent in both men and women with metabolic syndrome than those without. In all cities, mean CCAIMT and prevalence of plaque were higher in participants with metabolic syndrome than those without.

**Table 5:** Mean CCAIMT (mm), prevalence (%) and 95% confidence intervals, of carotid plaque according to the number of components of the metabolic syndrome by NCEP ATP III criteria in the CARMELA participants*

**Number of components**	**0**	**1**	**2**	**3**	**4**	**5**	**No metabolic syndrome**	**Metabolic syndrome**
Mean CCAIMT								
Men	0.623 (0.616-0.629)	0.644 (0.637-0.650)	0.665 (0.658-0.672)	0.688 (0.679-0.697)	0.707 (0.694-0.720)	0.731 (0.701-0.761)	0.645 (0.640-0.650)	0.695 (0.687-0.702)
Women	0.624 (0.617-0.632)	0.624 (0.618-0.631)	0.645 (0.639-0.652)	0.668 (0.662-0.675)	0.709 (0.696-0.721)	0.704 (0.685-0.723)	0.631 (0.626-0.636)	0.682 (0.676-0.688)
Prevalence of carotid plaque								
Men	4.0 (2.7-5.2)	7.2 (5.7-8.6)	7.8 (6.2-9.4)	9.3 (6.9-11.7)	9.7 (5.9-13.4)	11.4 (3.4-19.4)	6.4 (5.5-7.4)	9.5 (7.4-11.6)
Women	4.9 (3.5-6.2)	7.4 (5.9-8.8)	7.8 (6.3-9.4)	10.9 (8.5-13.3)	11.7 (8.1-15.4)	12.5 (6.2-18.8)	6.8 (5.9-7.7)	11.2 (9.3-13.2)

*CARMELA survey participants, including those with previous history of diabetes mellitus.

CCAIMT = carotid artery intima-media thickness

## Discussion

The CARMELA study reports overall prevalence of 21% of metabolic syndrome by NCEP ATP III criteria in the 7 Latin American cities studied. Profiles of the metabolic syndrome according to sex varied among cities. Among the overall population and the nondiabetic population, the highest prevalence of metabolic syndrome was found in Barquisimeto and Mexico City. Women of Bogota, Lima, Quito, and Santiago had greater prevalence of metabolic syndrome than their male counterparts, while the opposite was true in Buenos Aires. Not surprisingly, prevalence of metabolic syndrome increased with age, strikingly so in women. Participants with metabolic syndrome had higher mean CCAIMT and prevalence of carotid plaque than those without the syndrome.

The prevalence of metabolic syndrome found in CARMELA approximates prevalence estimates (23% to 27%) in the United States [6,13]. Direct comparison with other Latin America studies using NCEP ATP III criteria show CARMELA prevalence to be consistent with prevalence in Mexico [17,18]. Also by NCEP ATP III criteria, 31% of a population of Zulia State, Venezuela [19], and 12% and 26% of men and women of Lima [18], respectively, were found to have metabolic syndrome. CARMELA reports lower prevalence in Barquisimeto than elsewhere in Venezuela, and prevalence that is less disparate between the sexes of Lima; however, CARMELA targeted a much broader population base than in the aforementioned study in Lima. In the Estudio Peruano de Prevalencia de Enfermedades Cardiovasculares, or Peruvian Study of the Prevalence of Cardiovascular Risk Factors (PREVENCION) study in another urban region of Peru, 14% and 23% of men and women, respectively, were found to have the syndrome [20].

Whereas it has been suggested that the burden of metabolic syndrome is growing in younger populations, especially in developing regions [21], metabolic syndrome increases with age as CARMELA results confirm. Elevated body weight, waist circumference, and low HDL-C levels have been found to be more common contributors to metabolic syndrome in women, while blood pressure and apolipoprotein B have been found to be more common contributors in men [22]. Obesity in women is increasing faster than in men [23] and the metabolic syndrome increases in a parallel manner. In many women, menopause is accompanied by the emergence of features of the metabolic syndrome and increased cardiovascular risk, whether as a direct result of ovarian failure or indirectly related to central adipose redistribution [23,24]. With this constellation of factors, it has even been suggested that the metabolic syndrome should be defined by different criteria in men and women [22]. CARMELA results illustrate the amplified increase in metabolic syndrome in women as opposed to men as women reach menopausal age. A study of post-menopausal Ecuadorian women [25] revealed an overall prevalence of metabolic syndrome of 42%, similar to that found in CARMELA, with prevalence of hypertension, abdominal obesity, and low HDL-C similar to that in women in the older 2 CARMELA age groups. In a broader study of postmenopausal Latin American women, rates of metabolic syndrome in women of Buenos Aires were higher and those of Bogota, Lima, and Santiago, similar to those of women in the oldest 2 CARMELA age groups [26]. Although in all CARMELA cities, older women showed strikingly higher abdominal obesity rates than men, high rates of abdominal obesity were found in women of all age groups.

In designing targeted programs for detection and treatment of metabolic syndrome, CARMELA provides crucial data on the sensitivity and predictive value of each component. On the one hand, of the general population of men in CARMELA cities (except for Quito where lower prevalence of metabolic syndrome in men is noted), approximately 70% of those with glucose abnormalities, approximately two-thirds of those with abdominal obesity, and half with hypertension, will have the full syndrome. Of the general population of women in CARMELA cities, nearly three-quarters of those with glucose abnormalities, and approximately 50% to 60% with abdominal obesity, high triglycerides, or hypertension, will have the full metabolic syndrome (Table 2). On the other hand, once metabolic syndrome is diagnosed, the shape of the syndrome is characterized by predominance of hypertension, low HDL-C and high triglycerides in men, and predominance of abdominal obesity, high triglycerides, and a remarkable predominance of low HDL-C in women (Table 3). Thus, hypertension, low HDL-C, and high triglycerides in men and low HDL-C in particular, along with high triglycerides and abdominal obesity in women, are highly specific indicators of the syndrome. CARMELA provides data which confirms the likelihood of finding other specific components, once metabolic syndrome is diagnosed. Although the metabolic syndrome predicts diabetes along with cardiovascular risk, glucose abnormalities in and of themselves are not particularly specific to the full syndrome. This notion is confirmed by the similar prevalence of metabolic syndrome in CARMELA populations that both include and exclude diabetes. Since the metabolic syndrome is epitomized by the development of diabetes, its presence in the nondiabetic population is of particular importance.

To our knowledge, this is the first study that provides evidence of a relationship between the occurrence of the metabolic syndrome and the presence of carotid atherosclerosis in the Latin American population. A large study in Italy reported increased incidence of carotid plaques and stenosis over 5 years of follow-up in participants with metabolic syndrome [9]. Metabolic syndrome has also been associated with incident stroke in both black and white populations in the United States [6] and with higher carotid intima-media thickness and prevalence of plaques in elderly French individuals [27]. Metabolic syndrome has also been associated with tissue characteristics of the intima-media complex in the carotid artery [28], and the increasing number of components of the metabolic syndrome have also been related to higher carotid artery stiffness [29]. In other studies, the association between metabolic syndrome and carotid atherosclerosis has been reported to be stronger in women than men [30], however, in the CARMELA study it seems to be quite similar for men and women. Carotid intima-media thickening as an indicator of atherosclerosis in other vascular beds [31] is a strong predictor of future cardiovascular events [32]. The relationship between metabolic syndrome and carotid atherosclerosis noted in CARMELA enhances previous findings of metabolic syndrome as a cardiovascular disease predictor [3,7,9].

The value of CARMELA lies in its carefully constructed sampling of populations by age and sex to elucidate prevalence of metabolic syndrome components. Urban vs rural and ethnic differences in prevalence of metabolic syndrome have been reported in a variety of Latin American settings [33] and should be investigated further. Genetic predispositions to the metabolic syndrome [34], along with cultural and nutritional variations, also need investigation given Latin America's unique conglomeration of indigenous and immigrant populations. The obesity epidemic clearly affects the prevalence of metabolic syndrome among women but also extends to the young; CARMELA's lowest age range (25 to 34 years) may not have fully encompassed populations at risk, nor did CARMELA include increasingly older populations, which until now have borne the brunt of chronic disease. Rigorous studies like CARMELA should aid in designing targeted solutions to increasing rates of metabolic syndrome and its consequences.

In the Shape of the Nations Survey, 39% of participants worldwide who visited primary care physicians were overweight or obese, but only 58% of primary care physicians recognized that abdominal obesity was a significant risk factor for cardiovascular disease, and 45% reported that they never measured waist circumference [35]. As alarming as these statistics are, CARMELA findings reinforce the notion that metabolic syndrome is prevalent in urban Latin America and should be sought in the presence of even 1 component of the syndrome. Likewise, carotid atherosclerosis in Latin American individuals should spur healthcare providers to seek evidence of metabolic syndrome components and effect appropriate interventions. Guidelines for treatment of specific components in the context of the full metabolic syndrome are being developed [2] and should be promoted by healthcare educators and providers. The chronic disease burden consequent to metabolic syndrome will be extensive and expensive in Latin America if studies like CARMELA are ignored.

## Conclusion

CARMELA reports high prevalence of metabolic syndrome in 7 Latin American cities. Although rates varied between cities, there was a striking increase in the prevalence of metabolic syndrome with age, especially in women. CARMELA provides evidence for the association of metabolic syndrome with evidence of carotid atherosclerosis--namely increasing mean CCAIMT and plaque with increasing numbers of components of the syndrome, and overall higher mean CCAIMT and plaque in participants with the syndrome compared with those without. It is incumbent on government agencies and the medical community to address current prevalence of metabolic syndrome in order to prevent the dire consequences of increased burden of disease.

## Competing interests

RV is a permanent employee of Pfizer Inc, makers of a cholesterol-regulating drug, and has shares in the company. HS was an employee of Pfizer, Inc. during the conduct of the study (now retired). All other authors declare no competing interests.

## Authors' contributions

JE participated in the design and coordination of the study and drafted the manuscript. HSc conceived of the study, and participated in its design and coordination and helped to draft the manuscript. BC conceived of the study, and participated in its design and coordination and helped to draft the manuscript. HSi conceived of the study, and participated in its design and coordination and helped to draft the manuscript. CPB participated in the design and coordination of the study and helped to draft the manuscript. RV participated in the design and coordination of the study and helped to draft the manuscript. MT carried out the lab standardization. RH conceived of the study, and participated in its design and coordination and helped to draft the manuscript. EW conceived of the study, and participated in its design and coordination and helped to draft the manuscript.

All authors read and approved the final manuscript.

## Supplementary Material

Additional file 1 **Figure S1**. Prevalence of metabolic syndrome in each city, by age and sex. Figure S2. Mean CCAIMT and prevalence of plaque (95% Confidence Intervals), by city. Additional file 1 includes a couple of figures that help to understand data. Click here for file
