# Supplementary material for: Prevalence of the Metabolic Syndrome in Latin America and its association with sub-clinical carotid atherosclerosis: the CARMELA cross sectional study
Source: Cardiovasc Diabetol. 2009 Sep 26;8:52. doi: 10.1186/1475-2840-8-52 (PMC2760519; doi:10.1186/1475-2840-8-52)
Supplement: Additional file 1 — Figure S1. Prevalence of metabolic syndrome in each city, by age and sex. Figure S2. Mean CCAIMT and prevalence of plaque (95% Confidence Intervals), by city. Additional file 1 includes a couple of figures that help to understand data. [file 1475-2840-8-52-S1.DOC]

**Figure 1. Prevalence of metabolic syndrome in each city, by age and sex.**

**Figure 2. Mean CCAIMT and prevalence of plaque (95% Confidence Intervals), by city.**
